# CSF1R-Dependent Microglial Repopulation and Contact-Dependent Inhibition of Proliferation In Vitro

**DOI:** 10.3390/brainsci15080825

**Published:** 2025-07-31

**Authors:** Rie Nakai, Kuniko Kohyama, Yasumasa Nishito, Hiroshi Sakuma

**Affiliations:** 1 Department of Brain and Neuroscience, Tokyo Metropolitan Institute of Medical Science, Setagaya 156-8506, Japan; r55nakai@gmail.com (R.N.); kohyama-kn@igakuken.or.jp (K.K.); 2 Department of Pediatrics, Osaka University Graduate School of Medicine, Suita 565-0871, Japan; 3 Center for Basic Technology Research, Tokyo Metropolitan Institute of Medical Science, Setagaya 156-8506, Japan; nishito-ys@igakuken.or.jp

**Keywords:** microglia, repopulation, CD11b, colony-stimulating factor 1 receptor

## Abstract

Murine microglia exhibit rapid self-renewal upon removal from the postnatal brain. However, the signaling pathways that regulate microglial repopulation remain largely unclear. To address this knowledge gap, we depleted microglia from mixed glial cultures using anti-CD11b magnetic particles and cultured them for 4 weeks to monitor their repopulation ability in vitro. Flow cytometry and immunocytochemistry revealed that anti-CD11b bead treatment effectively eliminated >95% of microglia in mixed glial cultures. Following removal, the number of CX3CR1-positive microglia gradually increased; when a specific threshold was reached, repopulation ceased without any discernable rise in cell death. Cell cycle and 5-ethynyl-2′-deoxyuridine incorporation assays suggested the active proliferation of repopulating microglia at d7. Time-lapse imaging demonstrated post-removal division of microglia. Colony-stimulating factor 1 receptor-phosphoinositide 3-kinase-protein kinase B signaling was identified as crucial for microglial repopulation, as pharmacological inhibition or neutralization of the pathway significantly abrogated repopulation. Transwell cocultures revealed that resident microglia competitively inhibited microglial proliferation probably through contact inhibition. This in vitro microglial removal system provides valuable insights into the mechanisms underlying microglial proliferation.

## 1. Background/Objectives

Microglia, the resident macrophages of the central nervous system, originate from a cell lineage distinct from neurons and astrocytes. During cell turnover, terminally differentiated cells commonly exit the cell cycle, cease division, and are continually replenished through the proliferation of tissue-specific stem cells. However, the microglial population is uniquely sustained by the division of already differentiated cells. Previous studies using parabiotic mice and fate mapping analyses have revealed that adult microglia are primarily maintained through local self-renewal, with minimal contribution from circulating myeloid precursors [1,2].

The in vivo ablation of microglia leads to repopulation from the local expansion of residual microglia [3,4,5,6]. It has been hypothesized that CSF1R inhibitor-resistant cells which have progenitor-like features exist within heterogeneous subpopulations and contribute to repopulation [7]. At least under physiological conditions, adult microglia generate homogeneous subpopulations during the differentiation stage, and no progenitor cells have been identified thus far [8,9,10]. Several pathways involved in microglial repopulation have also been identified. CSF1R signaling is crucial for microglial proliferation during repopulation [3]. CX3CL1-CX3CR1 signaling was reported to be critical for microglial repopulation [11]. While P2RY12 receptors play a critical role in regulating the microglial landscape through cellular translocation, microglial repopulation is independent of P2RY12 receptor signaling [12,13]. Nevertheless, the mechanism driving the robust proliferation of adult microglia that is sufficient to recover from near-total ablation is not yet fully understood.

Although in vivo ablation methods are mostly employed for studying microglial repopulation [4], utilizing in vitro models of microglial repopulation holds an advantage as it facilitates real-time observation of the process and enables the assessment of responses to various interventions. In this study, we aimed to reveal the mechanisms of repopulation using in vitro microglial removal model.

## 2. Materials and Methods

### 2.1. Mice

Cx3cr1^*EGFP*/*EGFP*^ (B6.129P-Cx3cr1tm1Litt/J) mice, purchased from the Jackson Laboratory (Bar Harbor, ME), were inbred and maintained. Pregnant ICR and C57BL/6J wild-type mice were obtained from Japan SLC (Hamamatsu, Japan) and Charles River (Kanagawa, Japan), respectively. Cx3cr1^*EGFP*/*EGFP*^ male mice were crossed with C57BL/6J wild-type female mice to obtain Cx3cr1^+/*EGFP*^ mice. All mice were housed in a specific pathogen-free facility on a 12 h light and 12nh dark cycle with ad libitum access to food and water. All experiments were carried out in accordance with the National Institute of Health Guide for the Care and Use of Laboratory Animals revised in 1996. Formal approval to conduct the experiments was obtained by the Animal Care and Use Committee of the Tokyo Metropolitan Institute of Medical Science (approval code: 24-016; approval date: 1 April 2024).

### 2.2. Preparation of Primary Glial Culture

Primary mixed glial cultures were prepared from the brains of P 3–5 ICR or Cx3cr1^+/*EGFP*^ mice. Briefly, after the meninges were stripped off, the cerebral cortices were enzymatically (using 0.25% trypsin and 0.04% DNase-I) and mechanically (by homogenization and pipetting) dissociated. The cells obtained from 3–4 pups were suspended in Dulbecco’s Modified Eagle’s medium (DMEM, Nacalai Tesque, Kyoto, Japan) supplemented with 10% fetal bovine serum (FBS) and antibiotic–antimycotic mixed solution (1%, Nacalai Tesque) and were plated on a poly-L-lysine (Sigma-Aldrich, St. Louis, MO, USA)-coated 75 cm^2^ flask at 0.8–1 × 10^7^ cells/flask. The cells were maintained at 37 °C in a humidified atmosphere containing 5% CO_2_.

### 2.3. Removal of Microglia from Mixed Glial Culture Using Anti-CD11b Magnetic Particles

Within 7–12 days following the preparation of the primary glial culture (called mixed glial culture (MGC)), the cells were detached using TrypLE Express (1×) without phenol red (Thermo Fisher Scientific, Waltham, MA, USA). Subsequently, microglia were removed from the MGC using anti-mouse CD11b magnetic particles (BD Biosciences, Franklin Lakes, NJ, USA) according to the manufacturer’s protocol. Briefly, the detached cells per flask were resuspended in 50 µL of anti-CD11b magnetic particles and incubated at 4 °C for 40 min with gentle mixing every 5–10 min. Following conjugation with magnetic particles, the CD11b-positive fraction was removed using a BD DynaMag-15 Magnet (BD Biosciences). The isolated microglia-depleted cells or mixed glial cells were plated either in 24-well plates at a density of 1.0 × 10^5^ cells/well; in Lab-Tek II 8-chamber slides (Nagle Nunc International, Naperville, IL, USA) at a density of 1 × 10^5^ cells/well; in 35-mm, 4-compartment cell culture dishes with a glass bottom (Greiner Bio-One, Kremsmunster, Austria) at a density of 1.5 × 10^5^ cells/well; or in 48-well plates at a density of 1 × 10^5^ cells/well for subsequent experiments. The culture medium was replaced twice weekly.

### 2.4. Absolute Cell Counting by Flow Cytometry

Cells obtained from Cx3cr1^+/*EGFP*^ mice were harvested from 24-well plates using TrypLE Express (1×), without phenol red, at 4, 7, 11, 14, and 21 days after the removal of microglia. Subsequently, 5 µL of AccuCount fluorescent particles (Spherotech Inc., Lake Forest, IL, USA), at a known concentration of 1 × 10^6^ particles per mL, was added and analyzed using the LSRFortessa X-20 flow cytometer (BD, Franklin Lakes, NJ, USA). A total of 1000 particles were counted, and the absolute number of microglia per 1000 particles was determined. Data analysis was performed using FlowJo software ver10 (Tree Star, Ashland, OR, USA). Detailed gating strategies are provided in Supplementary Figure S1. This study adhered to the Guidelines for the Use of Flow Cytometry and Cell Sorting in immunological studies [14].

### 2.5. Microglial Surface Marker Analysis by Flow Cytometry

Seven days after removal, cells derived from Cx3cr1^+/*EGFP*^ mice were harvested from 24-well plates using TrypLE Express. The cells were treated with Fc Block (1:50, clone 93) and then stained with phycoerythrin (PE) anti-mouse CD11b (1:50, clone M1/70), allophycocyanin (APC) anti-mouse CD45 (1:50, clone 30-F11), PE anti-mouse F4/80 (1:50, clone BM8), APC anti-mouse P2ry12 (1:50, clone S16007D), PE anti-mouse Mertk (1:50, clone 2B10C42), and APC anti-mouse SiglecH (1:50, clone M1304A01) antibodies (all obtained from BioLegend, San Diego, CA, USA). Data were acquired on an LSRFortessa X-20 flow cytometer and analyzed using FlowJo software. The threshold event number was set to 10,000 cells in the CX3CR1-EGFP-positive gate. If the acquired cells did not reach this setting, a minimum of 2000 microglia were analyzed.

### 2.6. Confocal Microscopy

Fluorescent images were captured using an Olympus FV 3000 confocal microscope (Olympus, Tokyo, Japan). Image J software ver1.53 (NIH, Bethesda, MD, USA) was used to autonomically calculate the cell count, occupied area, and circularity of microglial cells (circularity = 4πS/L_2_). In brief, 8-bit grayscale images were converted to binary by setting a minimum threshold value of 50. The binary image underwent a despeckle process to eliminate small artifacts, and then particles (size 50-inf µm^2^) were analyzed using the “analyze particle” command. Circularity was calculated as 4π (area/perimeter^2^). The cells with a circularity close to 1 were considered to have a round morphology.

### 2.7. Microarray and Data Analysis

Single-color microarray analysis was performed to compare the gene expression levels between repopulating and control microglia obtained from ICR mice derived from mixed glial cultures 10 days after culture. Two samples from the control microglia (nos. 1 and 2) and two samples from the repopulating microglia (nos. 3 and 4) were prepared. Total RNA was purified from microglia using the RNeasy Mini Kit (QIAGEN, Hilden, Germany) according to the manufacturer’s instructions. RNA purity was assessed at absorbance ratios of 260 and 280 nm, with a ratio of 1.8–2.0 indicating good RNA purity. RNA was amplified and hybridized on the Agilent Whole Mouse Genome version 2.0 ST array (G4836A), washed, and scanned using a SureScan Microarray Scanner (Agilent Technologies, Santa Clara, CA, USA). The scanned images were analyzed using Feature Extraction software version 11.5.1.5 (Agilent Technologies). The genes were normalized to the 75th percentile intensity and clustered using GeneSpring GX12.9 (Agilent Technologies).

DEGs were considered significant if they exhibited an adjusted *p*-value of <0.05, determined using an unpaired *t*-test adjusted by the Benjamini–Hochberg method, with a 1.5-fold or greater change between the two samples (no. 1 vs. no. 3 and no. 2 vs. no. 4). Subsequently, an enrichment analysis was performed using the DAVID Gene Functional Classification Tool (link https://david.ncifcrf.gov/home.jsp, accessed on 24 January 2024) for DEGs, comparing repopulating microglia to controls.

### 2.8. Cell Cycle Analysis

Flow cytometry using DNA-selective, cell membrane-permeant staining was used for performing an indirect cell cycle analysis on days 4, 7, 11, 14, 22, and 28 after removal, following the manufacturer’s protocol. Briefly, microglia-depleted cultures or mixed glial cultures (control) from Cx3cr1^+/*EGFP*^ mice were detached from the 24-well plates. Cells were resuspended in Vybrant DyeCycle Violet stain (Thermo Fisher Scientific), which is excited at 405 nm and detected in the BV421 channel, diluted in DMEM without phenol red (Thermo Fisher Scientific) at a 1:1000 ratio. After gating GFP-positive cells, microglia in the G2/M phase were identified as BV421-high cells by flow cytometry, with the threshold determined using histogram-based gating.

### 2.9. EdU Cell Proliferation Assay

An EdU cell proliferation assay was performed using the Click-iT™ Plus EdU Alexa Fluor™ 647 Flow Cytometry Assay Kit (Thermo Fisher Scientific). Briefly, the cells from Cx3cr1^+/*EGFP*^ mice-derived microglia-depleted cultures or mixed glial cultures (control) were labeled with 10 μM EdU for 1 h. The EdU-incorporated cells were detached from the 24-well plate, fixed with paraformaldehyde for 5 min, permeabilized with a saponin-based buffer for 10 min, and treated with a Click-iT reaction cocktail containing Alexa Fluor 647 azide for 30 min. For analysis, microglia were first gated based on GFP. EdU-positive microglia were subsequently identified as Alexa Flour 647-high cells by histogram-based gating using flow cytometry.

### 2.10. Live Cell Imaging of Repopulating Microglia

Microglia-depleted cells and mixed glial cells from Cx3cr1^+/*EGFP*^ mice were plated and grown on 35 mm CELLview, a 4-compartment cell culture dish with a glass bottom (Greiner Bio-One), at a density of 1.5 × 10^5^ cells/well. At 7–12 days in vitro, the medium was replaced with FluoroBite DMEM (Thermo Fisher Scientific) supplemented with 10% FBS immediately before scanning. Confocal long-term live cell imaging of repopulating microglia was performed with a confocal scanner box (Cell Voyager CV1000, Yokogawa Electric Corp., Tokyo, Japan) from 5 to 8 days after removal (15 min intervals, 240 iterations, total of 60 h). Each well was scanned using a 10× objective lens in 8 randomly selected positions.

### 2.11. Propidium Iodide Staining Coupled with Flow Cytometry

Microglia-depleted cultures were prepared from ICR mice as described above. After 7 or 28 days of culture, the cells were detached from the 24-well plates using TrypLE Express (1×) without phenol red. The detached cells were incubated with Fc Block (1:50, clone 93) and stained with APC anti-mouse CD11b (1:50) (BioLegend) and PI (1:10) (BioLegend) according to the manufacturer’s protocol. PI-positive microglia were analyzed using flow cytometry.

### 2.12. Inhibition Experiments Against Csf1r Signaling

Microglia-depleted cultures were treated with the CSF1R inhibitor (PLX3397 (pexidartinib; Cayman Chemical, Ann Arbor, MI, USA), mouse CSF1R-blocking antibody (AFS98 (BioLegend)), MEK inhibitor (PD98059, Calbiochem, San Diego, CA, USA), PI3K inhibitor (LY294002, Tocris Bioscience, Bristol, UK), or Akt inhibitor (MK2206, Tocris Bioscience). The media were replaced with DMEM containing each inhibitor or CSF1R-blocking antibody twice a week, with a total of four replacements.

### 2.13. Enzyme-Linked Immunosorbent Assay for M-CSF in the Supernatant of Microglia-Depleted Cultures

Microglia-depleted cultures were prepared from B6J wild-type mice. Murine M-CSF concentrations in the supernatants of microglia-depleted cultures were measured via ELISA. A 96-well plate (Corning, Corning, NY, USA) was coated with 50 µL/well of capture Ab against M-CSF (PeproTech, Cranbury, NJ, USA) at 1:200 dilution and incubated overnight at 4 °C. Subsequently, the wells were washed three times with wash buffer (BD Biosciences, Franklin Lakes, NJ, USA) and blocked with 50 µL/well of Assay Diluent (AD) (BD Biosciences) for 1 h at RT. After removal of the blocking solutions and three washes, 50 µL/well of undiluted samples, standard solutions, and the negative control (AD) was added, and the plates were incubated at room temperature for 2 h. After three washes, 50 µL/well of detection Ab (PeproTech) was added at 1:400 dilution for 1 h at RT. After another three washes, 50 µL/well of Streptavidin-HRP was added at 1:1000 dilution. After five washes, 50 µL/well of substrate solution was added. When the standard solutions turned yellow, the reaction was stopped with 25 µL/well of stop solution (BD). The absorbance was measured at 450 nm using a 2030 ARVO^TM^X3 Multilabel Reader (PerkinElmer, Waltham, MA, USA).

### 2.14. Treatment with Recombinant M-CSF or M-CSF Blocking Antibody

MGC from Cx3cr1^+/*EGFP*^ mice were treated with recombinant mouse M-CSF (BioLegend) or mouse M-CSF blocking antibody (MAB4161, R&D Systems, Minneapolis, MN, USA). The medium was replaced with DMEM containing each compound twice a week, with a total of four replacements.

### 2.15. Coculture Experiments

Coculture experiments were performed using cultured cells derived from Cx3cr1^+/EGFP^ mice and ICR mice. Microglia-depleted cultures (i.e., “Dep”) derived from Cx3cr1^+/*EGFP*^ mice were cocultured with either Mgc or Dep derived from ICR mice, at 1:1 or 2:1 ratios. The cells derived from both Cx3cr1^+/*EGFP*^ mice and ICR mice were detached from the flasks; subsequently, the microglia were depleted (Dep), mixed at a 1:1 or 2:1 ratio, and seeded in 24-well plates. For non-contact coculture, ICR-derived cells were seeded separately from Cx3cr1^+/*EGFP*^ mice-derived cells using a Transwell^®^ insert with a 0.4 μm pore polycarbonate membrane (Corning). To assess whether secreted factors derived from mixed glial cultures suppress repopulation, the cells were cultured in the same volume of conditioned medium collected from independent mixed glial cultures which was replaced twice a week.

### 2.16. Statistical Analysis

All data are expressed as box-and-whisker plots, with the box depicting the median and the 25th and 75th quantiles and the whiskers showing the 5th and 95th percentiles. The line graph shows the mean values. Each experiment was independently repeated at least twice. Statistical analyses were performed using a two-tailed Welch’s *t*-test and one-way analysis of variance (ANOVA) and Turkey’s post test for multiple comparisons, as indicated. The Kolmogorov–Smirnov test was used to determine the normality of data. Analyses were performed using EZR [15] and a *p*-value of <0.05 was considered significant.

## 3. Results

### 3.1. Microglial Removal and Repopulation Using Anti-CD11b Magnetic Particles In Vitro

**Figure 1:**
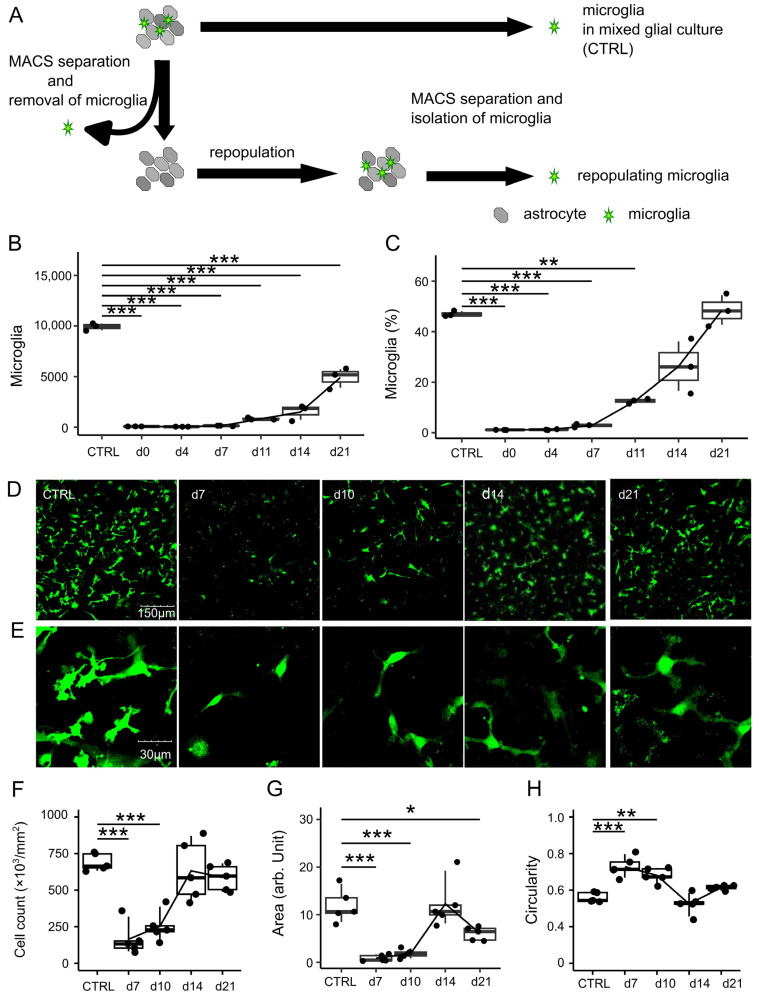
Microglial removal and repopulation in vitro. (**A**) Schematic diagram of microglial removal process in vitro. Mixed glial cultures were prepared from 3–5 Cx3cr1^+/*EGFP*^ or ICR pups. Microglia were removed from mixed glial cells using anti-CD11b magnetic particles (microglia-depleted culture). Repopulating microglia at each time point after removal were compared with microglia in mixed glial cell cultures at day 11 (CTRL). (**B**) Microglial cell counts per 1000 reference particles and (**C**) percentage of microglia in all cells were measured by flow cytometry for EGFP fluorescence of Cx3cr1^+/*EGFP*^ at indicated time points after removal. Statistics: one-way ANOVA. ** *p* < 0.01, and *** *p* < 0.001. Data are expressed as box-and-whisker plots, with all datapoints presented for three samples for each condition. Data are representative of two independent experiments. (**D**) Confocal microscopic images of CX3CR1-EGFP-positive microglia (green). Scale bars = 150 µm. (**E**) Higher magnification images show transient increase in less branched microglia on days 7–10. Scale bars = 30 µm. (**F**) Quantification of microglial cell counts per field, (**G**) area occupation, and (**H**) cell circularity at indicated time points were calculated. (**F**–**H**) Statistics: one-way ANOVA. * *p* < 0.05, ** *p* < 0.01, and *** *p* < 0.001. Data are expressed as box-and-whisker plots, with all datapoints presented for five samples per each condition. Data are representative of two independent experiments.

First, we evaluated the occurrence of microglial removal and subsequent repopulation in vitro. Microglia were removed from mixed glial cultures (MGCs) derived from Cx3cr1^+/*EGFP*^ mice using anti-CD11b magnetic particles (microglia-depleted cultures) (Figure 1A). After removal, the number and proportion of EGFP-positive microglia were serially measured by fluorescence-activated cell sorting (FACS) or microscopy. After cell death and doublet discrimination, the microglia identified as CX3CR1-EGFP-positive cells were quantified (Figure 1B,C, and Supplementary Figure S1). After the removal of >95% of microglia (*p* < 0.001 compared with control MGC) (day 0) through magnetic-associated cell sorting (MACS), the number of residual microglia rapidly increased on day 7. On day 21 after removal, the number of microglia recovered, but remained significantly lower than that in the control (*p* < 0.001) (Figure 1B). The percentage of microglia decreased to 1.13 (1.08–1.16) (*p* < 0.001) on day 0 and subsequently recovered to levels comparable to that of the control (*p* = 1.00) on day 21 (Figure 1C). Confocal microscopy revealed a marked decrease in the number of CX3CR1-EGFP-positive microglia (*p* < 0.001) upon removal but recovered to levels equivalent to that of the control (*p* = 0.94) and plateaued 2 weeks after removal (Figure 1D,F). Quantitative analysis revealed that the area occupied by microglia on day 7 significantly decreased compared with that of the control (*p* < 0.001) and recovered to the level equivalent to that of the control (*p* = 1.00) 2 weeks after removal (Figure 1G). Control microglia displayed multiple fine processes, while repopulating microglia exhibited transiently reduced branching and eventually returned to a ramified shape at 7–10 days after removal (Figure 1E). Morphological analysis indicated that the microglial circularity transiently increased to 0.66 ± 0.07 (*p* < 0.001) and 0.60 ± 0.05 (*p* = 0.002) at days 7 and 10, respectively, and returned to 0.40 ± 0.07 (*p* = 0.70) (Figure 1H). Time-lapse imaging showed that the dividing microglia, just before mitosis, transiently lost their processes and assumed a round shape (Supplementary Video S1). These results indicated that microglial removal is followed by repopulation, even in vitro, and that repopulating microglia exhibit transient morphological changes.

### 3.2. Signatures of Repopulating Microglia

**Figure 2:**
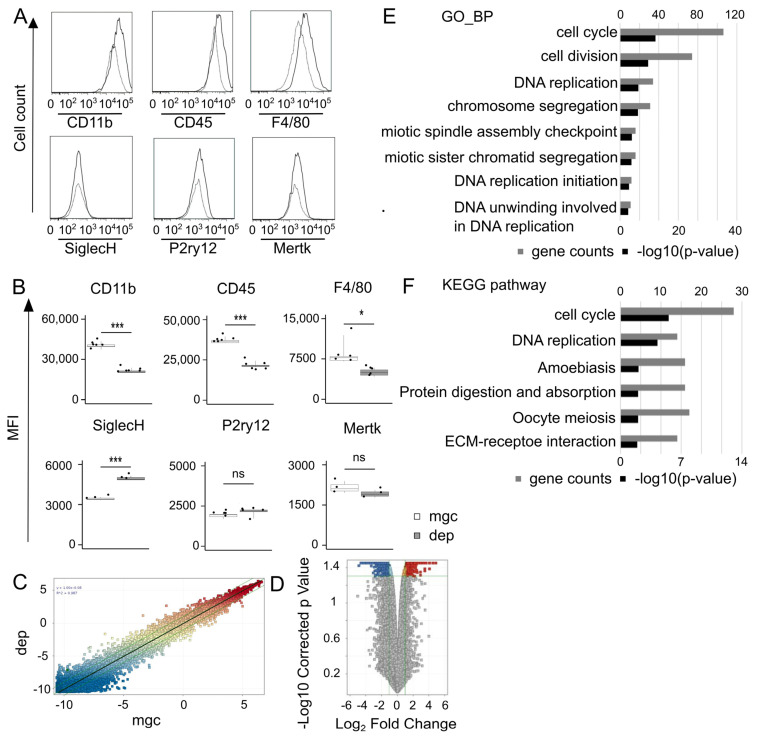
The signature of repopulating microglia. (**A**) The expression of surface markers on repopulating microglia (dotted line histograms) compared with CTRL (solid line histograms) on day 7 after removal analyzed by flow cytometry. (**B**) Mean fluorescence intensities of the microglial surface marker on CTRL and repopulating microglia. Data are expressed as box-and-whisker plots, with all datapoints presented for 3–6 samples per groups. Statistics: two-tailed *t*-test. * *p* < 0.05 and *** *p* < 0.001. ns, non-significant. Data are representative of three independent experiments. (**C**) Transcriptome analysis of CTRL and repopulating microglia on day 12. A scatter plot for DNA microarray analysis comparing repopulating microglia with CTRL. The axes of the scatter plot denote the normalized signal values of the samples. The thick green line indicates that the gene expression levels are the same for mgc and dep. The thin green lines indicate a 2-fold or greater change in expression levels between mgc and dep. (**D**) A volcano plot depicting fold change and *p*-value. Differentially expressed genes (DEGs) are defined as the genes with a *p*-value < 0.05 and a fold change value > 2.0 or <0.5. The blue dots denote down-regulated gene expression, the red dots denote up-regulated gene expression, and the gray dots denote the gene expression without marked differences. The horizontal line of the volcano plot represents the threshold (*p* < 0.05). The vertical dashed line of the volcano plot represents two-fold differences in expression. A total of 876 DEGs are shown in red (upregulated) or blue (downregulated) on the plot. (**E**) GO category analysis based on the biological process of upregulated genes. (**F**) KEGG pathway analysis of upregulated genes. (**E**,**F**) The upper horizontal axis shows genes counts, and the lower horizontal axis shows -log_10_ (*p* values). dep, microglia-depleted cultures; mgc, mixed glial cultures; DEGs, differentially expressed genes; GO, gene ontology; KEGG, Kyoto Encyclopedia of Genes and Genomes.

To investigate the signature of repopulating microglia in vitro, the expression of various molecules in microglia was analyzed using flow cytometry at 7 days after removal. The expression levels of CD11b, CD45, and F4/80 were significantly lower in the repopulating microglia compared with the controls (*p* < 0.001, *p* < 0.001, and *p* < 0.03, respectively) (Figure 2B). By contrast, the expression of SiglecH, a microglia-specific marker, significantly increased (*p* < 0.001) in repopulating microglia (Figure 2B). No significant differences were observed in the expression of other microglia-specific markers, including P2ry12 and Mertk (*p* = 0.15 and *p* = 0.24, respectively) (Figure 2A,B).

Microarray analysis comparing repopulating microglia and the control identified 876 differentially expressed genes (DEGs) (411 upregulated genes and 465 downregulated genes) (Figure 2C,D, Supplementary Tables S1 and S2). Gene ontology (GO) enrichment (Figure 2E) and Kyoto Encyclopedia of Genes and Genomes (KEGG) pathway analyses (Figure 2F) showed that the genes most enriched in repopulating microglia were associated with the cell cycle. These results suggest that repopulating microglia exhibit a relatively immature phenotype and enter the cell cycle. On the other hand, genes related to the interaction between extracellular matrix and receptors were mainly downregulated in the repopulating microglia (Supplementary Figure S2).

### 3.3. Proliferation and Cell Death During Repopulation

**Figure 3:**
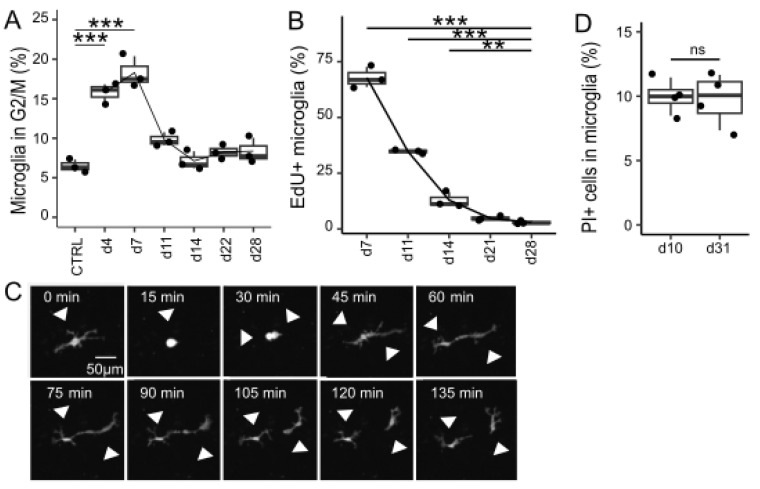
Proliferation and cell death during microglial repopulation. (**A**) Cell cycle analysis using DNA-selective staining during repopulation. The percentages of microglia in the G2/M phase (BV421 high) are shown. Control; mixed glial cell culture on day 11. Data are expressed as box-and-whisker plots, with all datapoints presented for three samples at each time point. Statistics: one-way ANOVA. *** *p* < 0.001. (**B**) EdU cell proliferation assay during repopulation. The percentages of EdU-positive microglia are shown. Data are expressed as box-and-whisker plots, with all datapoints presented for three samples at each time point. Statistics: one-way ANOVA. ** *p* < 0.01, and *** *p* < 0.001 as compared with day 28. (**C**) Representative montage of time-lapse images depicting repopulating microglia (grayscale). Arrowheads indicate microglial division. Scale bar = 50 μm. (**D**) Cell viability assay based on propidium iodide (PI) uptake. Microglia-depleted cultures derived from ICR mice were stained with APC anti-CD11b antibody/PI and were analyzed by flow cytometry. The percentage of PI-positive cells among the microglia was compared at day 10 and day 31 after removal. Data are expressed as box-and-whisker plots, with all datapoints presented for four samples. Statistics: two-tailed *t*-test. ns, non-significant. All data are representative of two independent experiments.

To elucidate the mechanisms underlying the increase in microglial count, their proliferation and cell death rates were evaluated. Cell cycle analysis revealed a significantly higher proportion of microglia in the G2/M phase compared with that in the control on days 4 (*p* < 0.001) and 7 (*p* < 0.001) after removal. On day 14, the proportion of microglia in the G2/M phase did not increase (*p* = 1.00) (Figure 3A). The EdU cell proliferation assay demonstrated a transient increase in the proportion of microglia labeled with EdU at 7, 11, and 14 days after removal compared with that at 28 days (*p* < 0.001, *p* < 0.001, and *p* = 0.01, respectively; Figure 3B). Time-lapse imaging confirmed the active division of microglia (Figure 3C and Supplementary Video S1). By contrast, the proportion of propidium iodide (PI)-positive cells among CD11b-positive microglia did not increase (*p* = 0.86) on day 31, when the number of microglia reached a plateau, compared with that on day 10 (Figure 3D). Taken together, these results suggest that the microglial proliferation rate temporarily increased after removal, leading to repopulation. Microglial proliferation was suppressed, while microglial death did not increase when the cell count reached a certain threshold.

### 3.4. Role of Colony-Stimulating Factor 1 Receptor-Phosphoinositide 3-Kinase-Protein Kinase B Signaling in Microglial Repopulation

**Figure 4:**
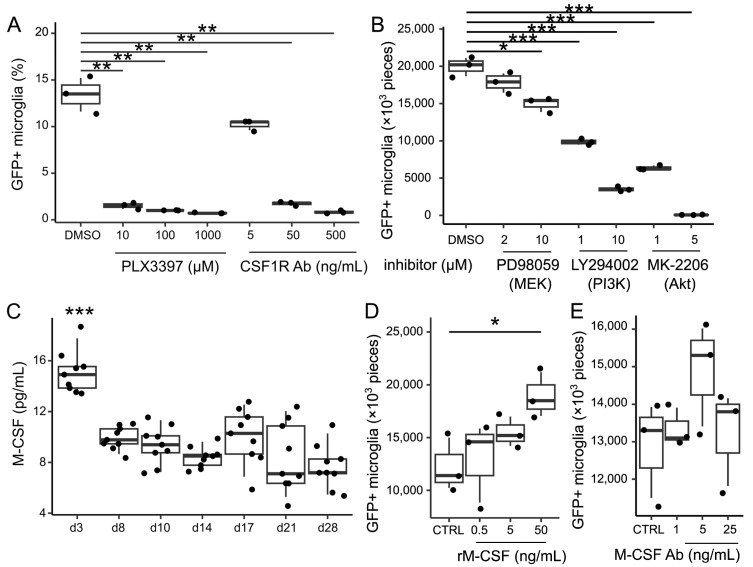
CSF1R-PI3K-Akt signaling is involved in microglial repopulation. (**A**) Flow cytometry analysis of Cx3cr1^+/*EGFP*^ mice-derived microglia-depleted cultures treated with a CSF1R inhibitor (PLX3397, 10, 100, or 1000 µM) or its antagonistic antibody (AFS98, 5, 50, or 500 ng/mL) at 2 weeks after microglia removal. Microglia-depleted culture treated by DMSO were used as a control. Data are expressed as box-and-whisker plots, with all datapoints presented for three samples in each condition. (**B**) Cx3cr1^+/*EGFP*^ mice-derived microglia-depleted cultures were treated with an MEK inhibitor (PD98059, 2 or 10 µM), PI3K inhibitor (LY294002, 1 or 10 µM), or Akt inhibitor (MK2206, 1 or 5 µM) for 2 weeks after microglia removal. Data are expressed as box-and-whiskers plots, with all datapoints presented for three samples. (**C**) ELISA of M-CSF in the supernatant from ICR mice-derived microglia-depleted culture during repopulation. Data are expressed as box-and-whisker plots, with all datapoints presented for nine samples. (**D**) Flow cytometry analysis of Cx3cr1^+/*EGFP*^ mice-derived microglia-depleted cultures stimulated with recombinant murine M-CSF (0.5, 5, or 50 ng/mL) at 2 weeks after microglia removal. (**E**) Flow cytometry analysis of Cx3cr1^+/*EGFP*^ mice-derived mixed glial culture treated with M-CSF-blocking antibody (1, 5, or 25 ng/mL) at 2 weeks after microglia removal. Data are expressed as box-and-whisker plots, with all datapoints presented for three samples. Statistics: two-tailed *t*-tests. * *p* < 0.05, ** *p* < 0.01, and *** *p* < 0.001. Data are representative of two independent experiments.

CSF1R signaling plays an important role in the survival and proliferation of microglia [3]. Hence, we examined the role of CSF1R signaling in the expansion of repopulating microglia. First, treatment with CSF1R inhibitors (PLX3397: 10, 100, and 1000 μM) or CSF1R-blocking antibodies (AFS98: 5, 50, and 500 ng/mL) on microglia-depleted cultures inhibited repopulation in a dose-dependent manner (Figure 4A). The chemical inhibition of the phosphoinositide 3-kinase (PI3K) or protein kinase B (Akt) pathways, which are downstream of CSF1R signaling, resulted in a decrease in the number of microglia, while MEK inhibition had no impact on microglial repopulation (Figure 4B). These results indicate that microglial repopulation is positively regulated by CSF1R-PI3K-Akt signaling.

Macrophage colony-stimulating factor (M-CSF) and interleukin (IL)-34 are the endogenous ligands of CSF1R. To verify the role of M-CSF in cell repopulation, the concentration of M-CSF in the supernatant was measured using the enzyme-linked immunosorbent assay (ELISA) after removal. The M-CSF concentration slightly increased to 14.9 (13.4–18.7) pg/mL (*p* < 0.001) on day 3 (Figure 4C). The concentration of IL-34, another ligand for CSF1R, was below the detection limit at all time points. Although treatment with 50 ng/mL of M-CSF increased the number of microglia compared with the control (*p* = 0.03), treatment with M-CSF at a concentration of 5 ng/mL or lower did not increase the number of repopulating microglia (Figure 4D). Further, treatment with M-CSF-neutralizing antibody (1, 5, and 25 ng/mL) did not significantly decrease the number of microglia (*p* = 0.61, *p* = 0.17, and *p* = 0.79, respectively) (Figure 4E). These results indicate that the concentration of M-CSF present in the culture supernatant is too low to increase the number of microglia, and much higher concentrations of M-CSF are required to induce microglial proliferation that can explain repopulation. Thus, a transient increase in the concentrations of M-CSF in the supernatant at day 3 does not account for microglial repopulation, and we speculated that microglial repopulation may be regulated by factors other than M-CSF.

### 3.5. Regulation of Microglial Repopulation Through Inhibition of Cell-to-Cell Contact

**Figure 5:**
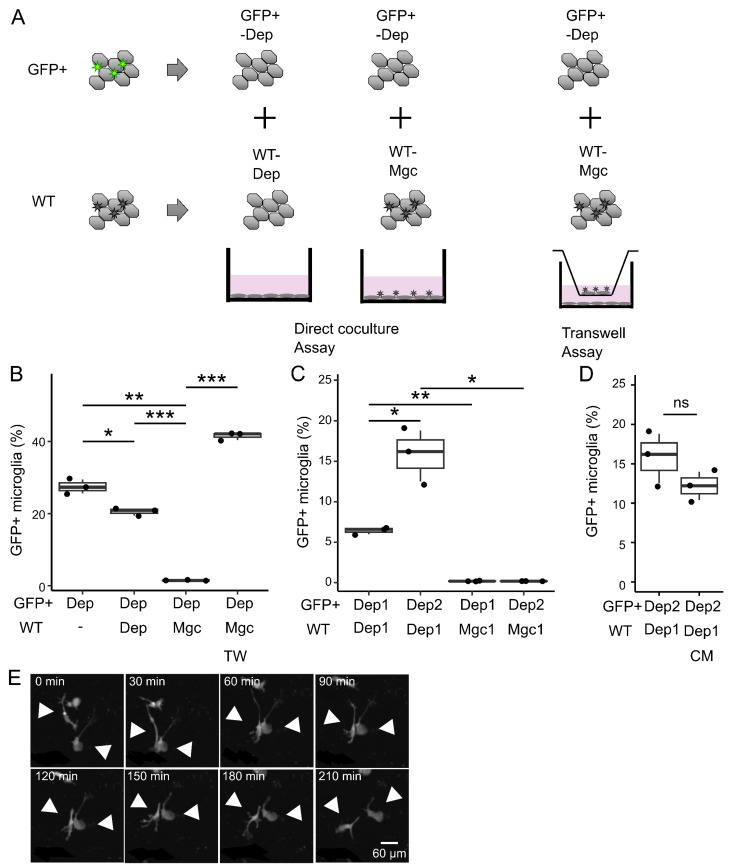
The effects of coexisting microglia or humoral factors on repopulation. (**A**) A schematic diagram of coculture assay. Cx3cr1^+/*EGFP*^ mice-derived microglia-depleted cells (GFP^+^-Dep) were cocultured with ICR mice-derived microglia-depleted cells (GFP^−^-Dep) or mixed glial cells (GFP^−^-Mgc). (**B**) The effect of coculture on the repopulation of Cx3cr1^+/*EGFP*^ mice-derived microglia. GFP^+^ microglia fractionation was counted at 28 days after coculture. (**C**) GFP^−^-Dep was cocultured with GFP^−^-Dep or GFP^−^-Mgc in different ratios. Dep2/Dep1 indicates that GFP^+^ and GFP^−^ cells were mixed in a 2:1 ratio. (**D**) The effect of mixed glial culture-conditioned media on the repopulation of Cx3cr1^+/*EGFP*^ mice-derived microglia. GFP^+^ microglia fractionation was counted at 14 days after coculture. CM, conditioned media. TW, Transwell. Data are expressed as box-and-whisker plots, with all data points representing the three samples. Data are representative of two independent experiments. Statistics: two-tailed *t*-tests. * *p* < 0.05, ** *p* < 0.01, and *** *p* < 0.001. ns, non-significant. Data are representative of two independent experiments. (**E**) A representative montage of time-lapse images depicting the direct contact of repopulating microglia (grayscale). Arrowheads indicate direct contact with microglia. Bar = 60 µm.

The number of microglia plateaued as their population increased. Thus, we hypothesized that the balance between proliferation and repression was maintained at a steady state, leading to a constant microglial density. To support this hypothesis, we investigated the potential factors inhibiting microglial growth and impeding their repopulation. To assess whether the existing microglia suppress the proliferation of microglia-depleted culture, we cocultured CX3CR1-EGFP (GFP^+^) mice-derived cells with ICR mice (GFP^−^)-derived cells (Figure 5A). First, the GFP^+^ microglia-depleted cultures were cocultured with GFP^−^ cells with or without microglia. The repopulation of GFP^+^ microglia was only suppressed when microglia were present in GFP^−^ cells (Figure 5B). However, the use of Transwell inserts to physically separate GFP^+^ cells from GFP^−^ microglia abolished the growth suppression effect (Figure 5B). Furthermore, the suppression of microglial repopulation persisted when GFP^+^ microglia-depleted cells and GFP^−^ mixed glial cells were mixed at a 2:1 ratio (Figure 5C). Furthermore, conditioned medium derived from mixed MGC did not suppress the microglial repopulation (Figure 5D), suggesting that growth suppression was not due to secreted factors. Time-lapse imaging revealed frequent contact between microglial cell bodies and processes (Figure 5E and Supplementary Video S2). These results may suggest that microglia negatively regulate the proliferation of neighboring microglia through an unknown mechanism, possibly through contact-dependent or paracrine mechanisms.

## 4. Discussion

Models of the transient removal of microglia using chemical or genetic methods have contributed significantly to elucidating the developmental origin and differentiation mechanisms of microglia, but also the role of microglia in various disease models. However, despite the valuable insights gained from these models, certain challenges persist and have not yet been effectively addressed. For example, an in vivo microglial ablation system, which induces massive cell death, has been reported to trigger a type 1 interferon-defined inflammatory cascade, possibly resulting in neuronal cell death [3,16]. The effects of such cell death have been a significant obstacle in evaluating the effects of microglial ablation on immune-mediated neurological diseases, including experimental autoimmune encephalomyelitis [16]. Furthermore, the release of damage-associated molecular patterns (DAMPs) from dying cells can induce proliferation and immune system activation in surrounding cells [17,18,19]. These complexities pose significant barriers to elucidating the detailed mechanisms governing microglial repopulation.

We deemed it crucial to employ physical rather than chemical methods to remove microglia, aiming to overcome challenges associated with existing models. In our approach, microglia were transiently removed from MGCs in vitro through magnetic separation. While chemical removal has the disadvantage that the effects of cell death mentioned above cannot be ignored, physical removal offers a solution to this problem even in vitro. Utilizing anti-CD11b magnetic particles in an in vitro microglia removal system, similar to the previously reported in vivo microglia ablation system [3,4,5,6], microglia were efficiently removed, initiating a subsequent repopulation process. In the in vitro setting, the repopulation process took more than 14 days, while microglia were reported to repopulate and return to baseline levels within 5–14 days after removal in vivo [3,4,5,20]. Compared with the in vivo method, the prolonged in vitro repopulation timeframe may be attributed to cell damage resulting from the cell detachment procedure and an environment less conducive to supporting microglial survival and proliferation in vitro. In addition, cell adhesion efficiency differs between glass and plastic bottoms, which results in slower cell proliferation on microscopic examination. Microglial repopulation did not persist and reached a plateau when the number of microglia was comparable to that of MGC.

Morphological assessments revealed transient changes in repopulating microglia, such as fewer branched processes and a more rounded soma, as reflected by an increase in microglial circularity within 7–10 days after removal. Similar rounded morphologies have been reported during the microglial development in vivo [21,22,23], reflecting a temporary state of immaturity. On day 21, the occupied area decreased, accompanied by an increase in circularity.

The decreased expression levels of CD11b, CD45, and F4/80 in repopulating microglia may also reflect immaturity. Conversely, the expression of microglia-specific markers, including P2ry12, Siglec-H, and Mertk, did not decrease. This indicates that repopulating cells retain their fundamental microglial characteristics regardless of their immaturity. In summary, repopulating microglia exhibited morphological and protein expression changes that transiently reflected their immaturity.

Even under physiological conditions, microglia maintain constant density through slow but stochastic proliferation and apoptosis [8,24]. Cell cycle analysis revealed that early repopulating microglia re-entered the cell cycle and exhibited increased proliferation. Transcriptome analysis supported this finding, indicating alterations in the expression of cell cycle-related genes in repopulating microglia. Time-lapse imaging further confirmed microglial division. In the time-lapse imaging, we were unable to observe the birth of CX3CR1-negative microglia, suggesting that, at least in this in vitro system, microglia are unlikely to be derived from CX3CR1-negative progenitors. This observation indicates that the local expansion of residual microglia contributes to repopulation [5,6].

Previous studies have shown that the oral administration of a CSF1R inhibitor in adult mice results in a 99% removal of microglia [3]. CSF1R signaling is essential for microglial survival and proliferation. Inhibition experiments using both chemicals and antibodies revealed that microglial repopulation is dependent on CSF1R signaling. Although the MEK-ERK and PI3K-Akt pathways are known to be downstream of CSF1R, microglial repopulation was unaffected by MEK-ERK inhibition but was significantly suppressed by either PI3K or Akt inhibition. Phosphorylation of PI3K and Akt was evaluated by Western blotting, but bands were not well detected. The essential role of CSF1R signaling is well known, and CSF1R-PI3K-Akt signaling is also deemed to be important for repopulation, albeit indirectly. In this model, neither M-CSF nor interleukin-34, the ligands for CSF1R, were added, suggesting that microglial proliferation was sustained by endogenous CSF1R ligands produced by unknown cells. Although M-CSF was identified in the culture supernatant, changes in its concentration could not account for the observed repopulation. It is reasonable to assume that the start of microglial repopulation is additionally regulated by factors other than the CSF1R-PI3K-Akt pathway.

We then examined the phenomenon by which microglia repopulation ceases when they are sufficiently proliferated. The cessation of repopulation was not due to an increase in microglial death, as the proportion of PI-positive cells did not increase when the number of microglia reached a plateau. Therefore, we hypothesized that an unknown mechanism may inhibit microglial proliferation. In direct contact cocultures of GFP^+^ and GFP^−^ cells, repopulation failed to occur when abundant coexisting microglia were present. This observation can be explained by a negative feedback mechanism in which microglia send inhibitory signals to each other, suppressing proliferation. The results from the experiments using conditioned media and Transwell assays support that signals inhibiting microglial proliferation are contact-dependent rather than mediated by secreted factors. Another possibility is that proliferation is inhibited by the transient, localized release of soluble factors when two microglial cells come into close proximity. In any case, such reciprocal growth inhibition could explain a variety of physiological features of microglia. In vitro, microglia have been observed to migrate, actively expand, and contract their processes. Although we did not assess the distance among microglia, microglia in the brain are arranged at regular intervals, suggesting that each cell occupies a territory within the brain parenchyma [8,24]. The growth inhibition against neighboring microglia may play a role in maintaining spatial homeostasis. Furthermore, the absence of known malignant tumors arising from microglia suggests an extremely potent growth suppression mechanism within these cells.

It is also known that the ability of microglia to repopulate is not infinite. If the removal of microglia was repeated in short cycles (7 days’ treatment and 7 days’ recovery) using a CSF1R inhibitor, the microglia no longer repopulated after the second removal [25]. Unsolved questions remain regarding microglial repopulation and await elucidation of the sensing mechanism of microglial depletion.

This study has several limitations. Firstly, microglia undergo rapid changes in their properties, giving rise to heterogeneous subpopulations when examined ex vivo [25]. Thus, it is unclear whether the observations in the present study accurately reproduced the phenomena occurring in microglia in vivo. Additionally, the current in vitro culture system differs from the living brain environment as it primarily consists of microglia, astrocytes, and oligodendrocytes, and does not include neurons or vasculature. Secondly, we used different strains of mice in different experiments, and it is possible that this difference affected the results in some experiments. However, since microglial repopulation was observed in the same manner in the two different strains of mice, we considered it unlikely that differences in mouse strain would seriously affect the outcome of the co-culture. Thirdly, we did not provide direct evidence that PI3K–AKT activation specifically occurs downstream of CSF1R signaling in the context of microglial proliferation during repopulation. The identification of the master regulators of microglial proliferation remains challenging.

## 5. Conclusions

We used an in vitro microglial removal system followed by repopulation. Using this experimental system, we elucidated several mechanisms regulating microglial repopulation. Despite these limitations, in vitro microglial removal remains a promising approach for investigating microglial proliferation.

## Data Availability

The datasets generated and analyzed in the present study are available from the corresponding author upon reasonable request. The datasets are not available publicly due to time limitation.

## Supplementary Materials

The following supporting information can be downloaded at https://www.mdpi.com/article/10.3390/brainsci15080825/s1: Supplementary Figure S1. (A) Gating strategy for flow cytometry analysis of CX3CR1-EGFP-positive microglia. Dead cells and counting particles were excluded. Live cells were gated on forward (FSC) versus side scatter (SSC). Next, doublets were excluded. Finally, CX3CR1-EGFP-positive microglia were gated. (B) Gating strategy for PI-positive microglia. After CD11b+ microglia were extracted as in (A), PI-positive cells in microglia were gated; Supplementary Figure S2. (A) GO category analysis based on the biological process of downregulated genes. (B) KEGG pathway analysis of downregulated genes. (A,B) The upper horizontal axis shows gene counts, and the lower horizontal axis shows -log_10_ (*p* values). GO, gene ontology; KEGG, Kyoto Encyclopedia of Genes and Genomes; Supplementary Table S1. A list of the upregulated (1) or downregulated (2) genes detected in the microarray analysis. Genes with a *p*-value < 0.05 and a fold change >2.0 or <0.5 that passed the filter were extracted; Supplementary Table S2. DAVID enrichment analyses were applied only to these differentially expressed genes; Supplementary Video S1. Time-lapse imaging of repopulating microglia recorded 5–8 days after removal revealed microglia that transformed into a round shape and underwent cell division. Interval = 15 min; Supplementary Video S2. Time-lapse imaging of repopulating microglia was recorded 5–8 days after removal, showing direct contact between the microglia. Interval = 15 min.

## Abbreviations

The following abbreviations are used in this manuscript:

MGC	Mixed glial culture
DMEM	Dulbecco’s Modified Eagle’s medium
FBS	Fetal bovine serum
PE	Phycoerythrin
APC	Allophycocyanin
DEGs	Differentially expressed genes
CSF1R	Colony-stimulating factor 1 receptor
FACS	Fluorescence-activated cell sorting
MACS	Magnetic-associated cell sorting
GO	Gene Ontology
KEEG	Kyoto Encyclopedia of Genes and Genomes
PI3K	Phosphoinositide 3-kinase
Akt	Protein kinase B
M-CSF	Macrophage colony-stimulating factor
ELISA	Enzyme-linked immunosorbent assay
DAMPs	Damaged-associated molecular patterns
