# Supplementary figures and images for: CSF1R-Dependent Microglial Repopulation and Contact-Dependent Inhibition of Proliferation In Vitro
Source: Brain Sci. 2025 Jul 31;15(8):825. doi: 10.3390/brainsci15080825 (PMC12384364; doi:10.3390/brainsci15080825)

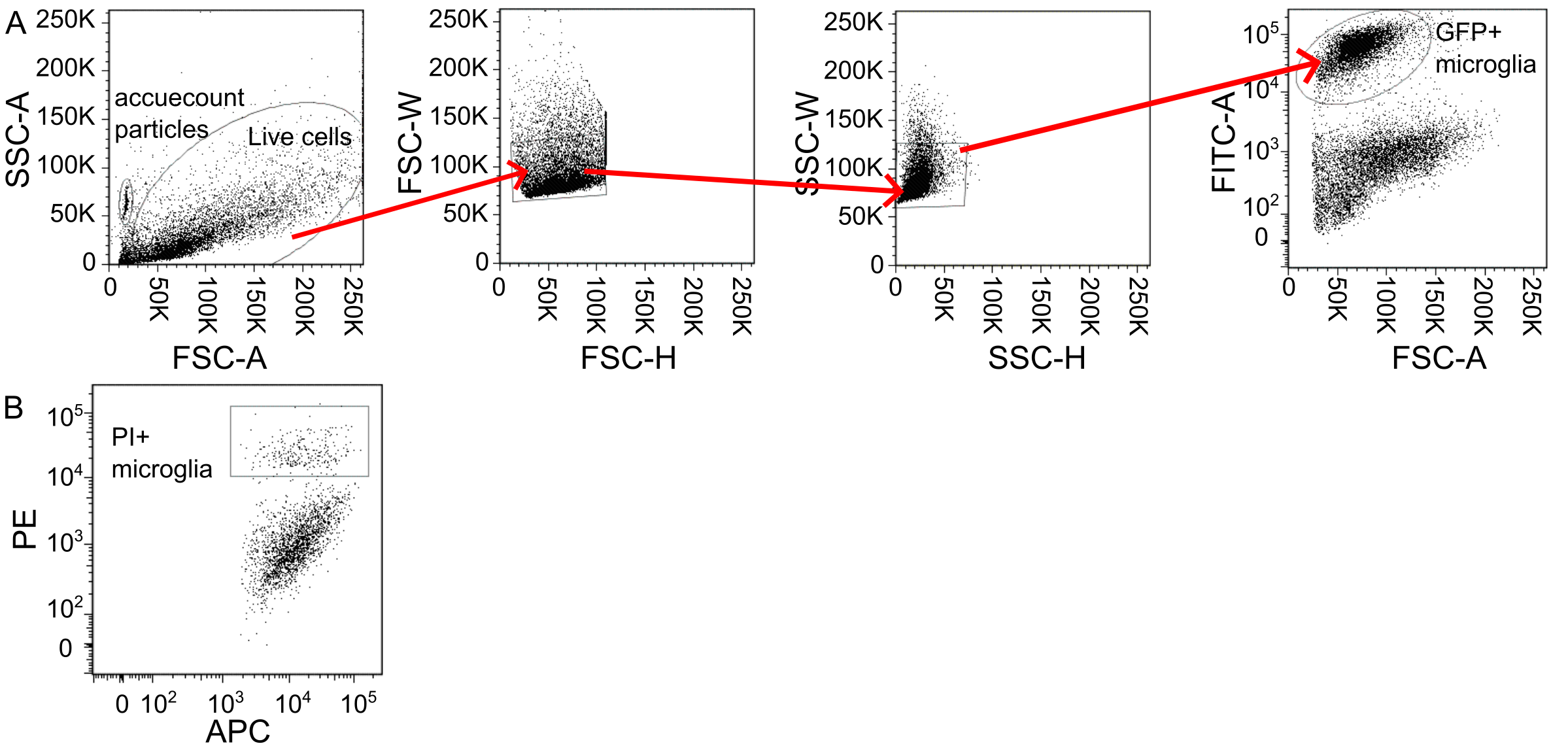

Supplement: Supplementary file 1 [file brainsci-15-00825-s001.zip › supplementary Fig S1_174mm.png]
